# Can lung semi-quantitative measurements and mediastinal adipose tissue volume predict prognosis in patients with idiopathic pulmonary fibrosis (IPF)? A CT-based preliminary study

**DOI:** 10.5578/tt.20239702

**Published:** 2023-09-22

**Authors:** H. AKKAYA, Ö. ERÇEN DİKEN

**Affiliations:** 1 Clinic of Radiology, University of Health Sciences, Adana City Training and Research Hospital, Adana, Türkiye; 2 Clinic of Chest Diseases, University of Health Sciences, Adana City Training and Research Hospital, Adana, Türkiye

**Keywords:** Mediastinal adipose tissue volume, IPF, HRCT, semi-quantitative analysis, Mediastinal yağ doku hacmi, İPF, YÇBT, yarı-kantitatif ölçüm

## Abstract

**ABSTRACT:**

Can lung semi-quantitative measurements and mediastinal adipose tissue volume predict prognosis in patients with idiopathic pulmonary fibrosis (IPF)? A CT-based preliminary study

**Introduction:**

The aim of this study was to assess the potential of subcutaneous adipose tissue volume, mediastinal adipose tissue volume, lung density, and lung volume (as measured on high-resolution computed tomography) to predict disease progression in patients with idiopathic pulmonary fibrosis (IPF). Additionally, the study aimed to evaluate the changes in these semiquantitative measures over time.

**Materials and Methods:**

The HRCT images of 57 patients diagnosed with IPF were retrospectively screened. Subcutaneous adipose tissue volume, mediastinal adipose tissue volume, and mean lung density and volume were measured at the time of diagnosis and at the 12th month. The ability of these parameters to predict progression was evaluated using the univariate and multivariate Cox regression analyses.

**Results:**

Low mediastinal adipose tissue volume at diagnosis had a 0.991-fold effect [odds ratio (OR)= 0.991, 95% confidence interval (CI)= 0.984-0.997, p< 0.001] on progression. Low mediastinal adipose tissue volume at diagnosis had a 0.993-fold effect [odds ratio (OR)= 0.993, 95% confidence interval (CI)= 0.975-1.011, p< 0.001] and progression development at the 12th month had a 6.5-fold effect [odds ratio (OR)= 6.516, 95% confidence interval (CI)= 1.594-26.639, p< 0.009] on mortality.

**Conclusion:**

This study indicate that the prognosis was better in those with a large mediastinal adipose tissue volume among the patients with IPF.

## Abstract (tr)

**ÖZ:**

Yoğun bakımdaki yaşlı kırılgan hastalarda diyafram ultrasonu mekanik ventilatörden ayrılmayı hızlı yüzeyel solunum indeksine göre daha iyi öngördürebilir mi?

**Giriş:**

Bu çalışmanın amacı, yüksek çözünürlüklü bilgisayarlı tomografide (YÇBT) ölçülen deri altı yağ dokusu hacminin, mediastinal yağ dokusu hacminin, akciğer dansitesinin, akciğer hacminin ve bu yarı kantitatif ölçümlerin zaman içindeki değişiminin idiyopatik pulmoner fibrozlu (İPF) hastalarda hastalığın ilerlemesini öngörmede kullanılıp kullanılamayacağını değerlendirmektir.

**Materyal ve Metod:**

İdiyopatik pulmoner fibroz tanısı alan 57 hastanın YÇBT görüntüleri retrospektif olarak tarandı. Tanı anında ve 12. ayda cilt altı yağ dokusu hacmi, mediastinal yağ dokusu hacmi ve ortalama akciğer yoğunluğu ve hacim değerleri ölçüldü. Bu parametrelerin ilerlemeyi tahmin etme yeteneği, tek değişkenli ve çok değişkenli Cox regresyon analizleri kullanılarak değerlendirildi.

**Bulgular:**

Tanı anındaki düşük mediastinal yağ dokusu hacminin progresyon üzerinde 0,991 kat [odds oranı (OR)= 0,991, %95 güven aralığı (CI)= 0,984-0,997, p< 0,001] etkisi vardı. Tanı anındaki düşük mediastinal yağ doku hacminin mortalite üzerinde 0,993 kat [odds oranı (OR)= 0,993, %95 güven aralığı (CI)= 0,975-1,011, p< 0,001] etkisi ve 12. ayda gelişen progresyonun mortalite üzerinde 6,5 kat [odds oranı (OR)= 6,516, %95 güven aralığı (CI)= 1,594-26,639, p< 0,009] etkisi vardı.

**Sonuç:**

Bu çalışma, tanı anında mediastinal yağ dokusu daha fazla olan İPF’li hastalarda prognozun daha iyi olduğunu göstermiştir.

## Introduction

The diagnosis of idiopathic pulmonary fibrosis (IPF) is made on the basis of a combination of data from clinical history, laboratory tests, radiological imaging, and, more rarely, pathological findings ( 1 ). Histopathological hallmarks of IPF include a combination of four features: 1) patchy dense fibrosis with architectural distortion; 2) predilection for subpleural or paraseptal lung parenchymal; 3) presence of fibroblastic foci and 4) absence of traits that would suggest an alternative diagnosis. The most common changes identified with high-resolution computed tomography (HRCT) in IPF are reticular densities, fibrosis, honeycomb appearance, and traction bronchiectasis ( 2 , 3 ). Similarly, the increase in the prevalence of these defined lesions is the most common indication in the diagnosis of progression ( 3 , 4 ). The identification of IPF has clinical implications since these diseases are associated with impaired respiratory function, risk of progression, and consequently increased mortality ( 4 , 5 , 6 ). Therefore, we aimed to investigate whether the tomography findings at the time of diagnosis of IPF patients have an effect on the prognosis of the disease and the risk of mortality ( 5 , 6 ). HRCT evaluation by radiologists can provide a prognostic prediction, but no standard method or consensus is available for this purpose, and therefore there is still a need for new criteria with high sensitivity and specificity ( 3 , 6 , 7 ).

This study aimed to evaluate the prognostic predictive value of semi-quantitative data obtained from the HRCT examination of patients with IPF, including lung density and volume, as well as mediastinal and subcutaneous adipose tissue volumes.

## MATERIALS and METHODS

### Patient Selection and Study Design

This study was approved by the ethics committee and conducted in full accordance with the guidelines of the Declaration of Helsinki. The requirement for informed consent from the patients was waived due to the retrospective nature of the study.

The HRCT images of 168 patients diagnosed with idiopathic interstitial fibrosis (IPF) and followed up in our hospital between January 2017 and May 2022 were screened. The diagnosis of the patients was made in accordance with the international IPF diagnostic criteria ( 6 , 7 , 8 ). The patients included in the study were those who were diagnosed with IPF in collaboration with the clinician and radiologist, taking into account clinical findings, HRCT findings, pulmonary function, and carbon monoxide diffusion test results. Only patients with defined usual interstitial pneumonia (UIP) HRCT findings were included in the study. All the patients were diagnosed with IPF after excluding other factors through clinical history and laboratory tests. Therefore, these patients did not necessitate a pathological diagnosis. A multidisciplinary discussion (MDD) was conducted for each patient, which integrated clinical, radiological, physiological, and laboratory data and findings.

The diagnosis of progression was established based on the presence of two out of the three criteria: worsening symptoms, radiological progression, and physiological progression ( 7 ). Radiological progression criteria included the presence of increased reticular opacity, thickening of bronchovascular bundles, an increased number of existing traction bronchiectasis or newly emerging traction bronchiectasis, and an expansion of honeycomb areas in IPF patients ( 1 , 2 , 5 , 7 ). Physiological progression criteria encompassed reductions in the diffusing capacity of the lungs for carbon monoxide (DLCO), the ratio of diffusing capacity to alveolar volume (DLCO/VA), and forced vital capacity (FVC). Patients were prescribed pirfenidone or nintedanib based on clinical and laboratory values, as determined by the clinician’s discretion ( 5 , 6 , 8 ). All patients exhibited a definite UIP pattern in HRCT and received a clinical diagnosis of (IPF after excluding other pathological conditions. Patients with additional comorbidities (such as pulmonary hypertension, ischemic heart disease, lung cancer, emphysema/chronic obstructive pulmonary disease, gastroesophageal reflux, and sleep apnea) in their medical history that may affect mortality were not included in the study. All patients received their initial diagnosis at our hospital.

Patients who experienced pneumonia during the follow-up period, tested positive for COVID-19 via PCR, exhibited clinical or radiological (thoracic computed tomography) signs suggestive of COVID- 19, discontinued follow-up, or had a history of surgery were excluded from the study ( Graphic 1 ). In addition, six patients with pneumothorax were excluded since this condition could affect the density and volume measurements. Following the application of the study criteria, a total of 69 patients who met any of these exclusion criteria were removed from the study, leaving 57 patients eligible for evaluation, and they were included in the study.

**Graphic 1:**
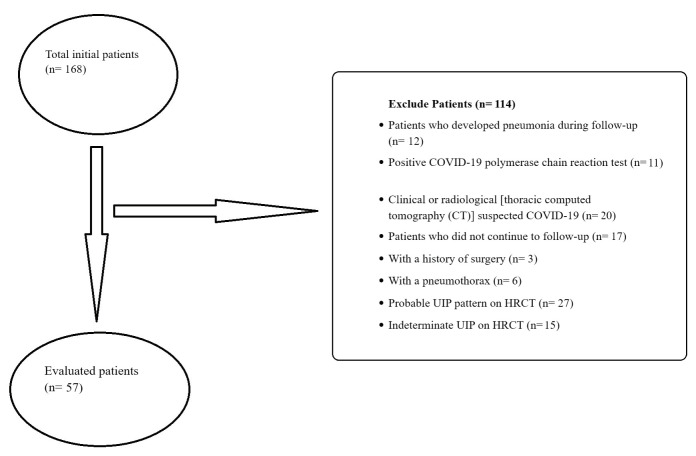
The initial total number of patients, along with the count of patients enrolled in the study, is presented. The graph also illustrates the reasons for exclusion from the study.

### HRCT Acquisition

HRCT scans were performed using a 128-detector scanner (Philips Ingenuity 128; Philips, Eindhoven, the Netherlands). All scans were completed during a single breath-hold while the patients were in the supine position. The standard scanning area was designated as the space between the apex of the lungs and the costophrenic angles. The following CT parameters were used: tube voltage, 120 kVp; tube current, 100-200 mAs; gantry rotation time, 0.5 s; pitch, 0.8 or 1; slice thickness, 1 mm; and slice reconstruction, 3 mm. All the semi-quantitative measurements were performed using Philips IntelliSpace Service Healthcare workstations.

### CT Evaluation

**Figure 1:**
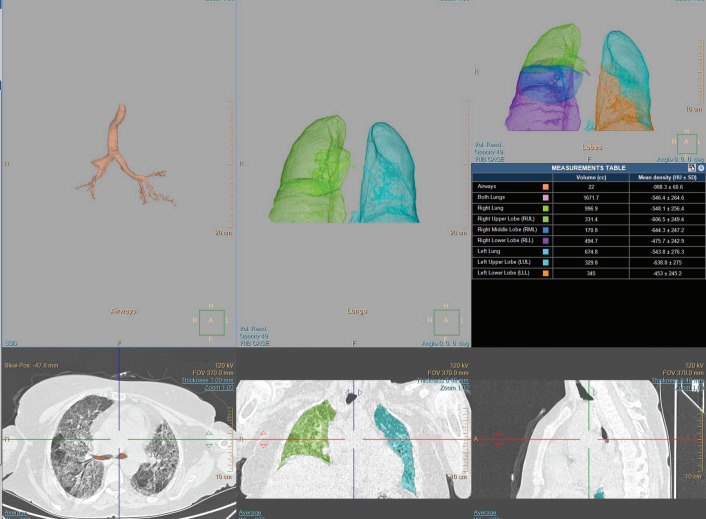
Lung density and lung volume measurements performed automatically at the workstation.

Automatic quantification of lung CT density was employed to identify the proportion of lung voxels with high-attenuation areas, typically between -800 and -250 Hounsfield units (normal CT attenuation of the lung was considered to be approximately -750 Hounsfield units) ( 2 , 3 , 6 ) ( Figure 1 ).

**Figure 2:**
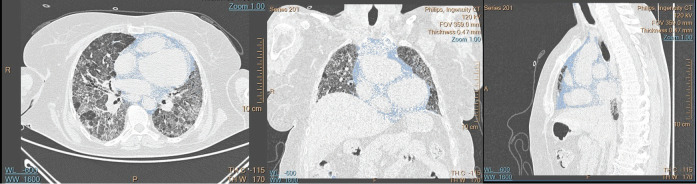
Measurement of mediastinal adipose tissue volume as observed in axial, coronal, and sagittal planes of HRCT.

Following the study protocol, the measurement of subcutaneous adipose tissue volume in HRCT scans was conducted by manually identifying the level from the superior thoracic aperture to the costodiaphragmatic recess. This was achieved using the sub-selection method within the workstation (Philips IntelliSpace). Subsequently, the volume of adipose tissue up to this identified level was calculated. In the female patients, breast tissue was calculated separately and extracted from the total adipose tissue volume. The HRCT parenchyma window was used during all the measurements and evaluations. A range of -130 to -30 HU was used for subcutaneous and mediastinal adipose tissue voxels, with reference to previous studies in the literature ( 3 , 5 , 6 ) ( Figure 2 ).

During the measurement process, all images generated by the software were meticulously reviewed by two radiologists to identify any potential errors. If needed, these radiologists manually implemented corrections to ensure accuracy. Furthermore, manual corrections were executed using the “editing tool” to prevent the inclusion of solid organs, intestines, vessels, and fat-free tissues such as the skeleton within the measurement areas. The subcutaneous, and mediastinal adipose tissue volumes were automatically measured in milliliters by the software. All measurements were conducted by two radiologists, reaching a consensus in their assessments. Their interpretations were not blinded to each other’s observations. All measurements were made semi-quantitatively. No visual comments were made. For the measurements, all the images obtained from the software were used. No extrapolation procedures, such as addition and multiplication were utilized, which allowed for the calculation of the actual adipose tissue volumes.

### Statistical Analysis

The Statistical Package for the Social Sciences (SPSS) v25.0 software package was used for the statistical analysis of the data. Categorical measurements were summarized as numbers and percentages, and continuous measurements as mean and standard deviation values (median and minimum-maximum where appropriate). The Chi-square and Fisher’s exact tests were used when comparing categorical variables. The Shapiro-Wilk test was conducted to determine whether the parameters investigated in the study showed a normal distribution. The independent Student’s t-test was used for normally distributed parameters and the Mann-Whitney U test for non-normally distributed parameters. The sensitivity and specificity values of the subcutaneous adipose tissue volume, mediastinal adipose tissue volume, and lung density at the time of diagnosis as well as lung volume at first examination were calculated for the prediction of progression, and those of the initial mediastinal adipose tissue volume were calculated for the prediction of mortality. The changes from the initial to the 12th-month measurements were expressed using ∆. In addition, the area under the receiver operating characteristic (ROC) curve was examined, and the cut-off values were determined. The established Cox regression model included patients’ gender, age, progression at 12 months, and initial subcutaneous adipose tissue volume, mediastinal adipose tissue volume, lung density, and lung volume. In the created model, the relevant variables were primarily examined in the univariate analysis. The statistical significance level was taken as 0.05 in all the tests.

## RESULTS

**Figure 3:**
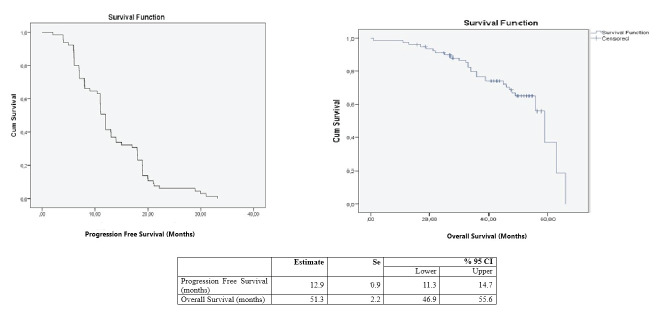
Progression-free survival and overall survival times of patients.

In this study, a total of 57 patients with IPF were retrospectively evaluated ( Table 1 ). The progressionfree period of the patients was 12.9 months, and the overall survival time was 51.3 months (Figure 3). Mortality was found to be higher in patients with progression (p< 0.005). The patients with progression had lower initial and 12th-month mediastinal adipose tissue volume, ∆ mediastinal adipose tissue volume, ∆ lung density values, and higher ∆ lung volume values compared to those without progression (p= 0.014). No significant differences were observed in the remaining parameters listed in Table 2 based on progression development (p> 0.05). No correlation was identified between the patients’ weights, body mass indices, and the volume of mediastinal adipose tissue, as shown in Table 3 .

Gender, age, initial subcutaneous adipose tissue volume, mediastinal adipose tissue, lung density, and lung volume values were included in the Cox regression model. In the created model, the relevant variables were primarily examined with the univariate analysis. According to the results, a low mediastinal adipose tissue volume at the time of diagnosis had a 0.993-fold [odds ratio (OR)= 0.993, 95% confidence interval (CI)= 0.987-0.999, p< 0.001] effect on progression. As a result, it was determined that only a low mediastinal adipose tissue volume at the time of diagnosis had a 0.991-fold (OR= 0.991, 95% CI= 0.984-0.997, p< 0.001) effect on progression (p< 0.05) ( Table 4 ).

Based on the examination results, the development of progression at 12 months exhibited an 8.4-fold impact on mortality. The findings further indicated that only the progression development at the 12th month had a 6.5-fold impact on mortality (OR= 6.516, 95% CI= 1.594-26.639, p< 0.009) on mortality ( Table 5 ).

**Figure 4:**
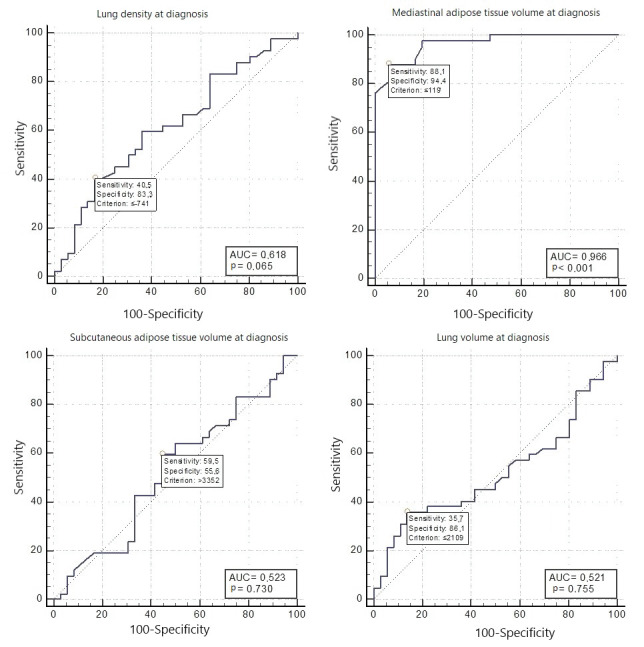
Receiver operating characteristic (ROC) curve illustrating the diagnostic efficacy of HRCT measurements at the time of diagnosis in predicting disease progression.

The results revealed that the mediastinal adipose tissue volume at the time of diagnosis had a diagnostic test success of 96.6%, with a mediastinal adipose tissue volume value below 119 mL having a sensitivity of 88.1% and specificity of 94.4% in identifying the patients with progression (p< 0.001). The mean lung density and lung volume at the time of diagnosis had very low sensitivity in predicting progression, and their diagnostic test performance was not statistically significant ( Figure 4 ).

**Table 1:** Demographic data of the study group and semi-quantitative values measured on HRCT

	Frequency (n)	Percentage (%)
Gender		
Female	22	38.6
Male	35	61.4
Progression		
Absent	23	40.4
Present	34	59.6
Mortality		
Absent	39	68.4
Present	18	31.6
Progression at month 12		
Absent	15	26.3
Present	42	73.7
	Mean ± SD	Median (min-max)
Age, years	56.9 ± 8.7	58.5 (37-71)
Height (cm)	170.2 ± 5.4	169.5 (157-181)
Weight (kg)	71.7 ± 4.8	72.5 (61-84)
BMI (kg/m2)	24.7 ± 2.2	24.6 (19.7-30.4)
Subcutaneous adipose tissue volume at diagnosis (mL)	3433.7 ± 913.7	3431.5 (998-5616)
Mediastinal adipose tissue volume at diagnosis (mL)	135.9 ± 48.7	120 (67-312)
Lung density at diagnosis (HU)	-686.8 ± 82.5	-678 (-876 - -474)
Lung volume at first examination (mL)	2797.9 ± 1191.6	2602 (716-5747)
Subcutaneous adipose tissue volume at month 12 (mL)	3511.1 ± 932.8	3545.5 (1135-5552)
Mediastinal adipose tissue volume at month 12 (mL)	143.4 ± 51.8	128 (69-326)
Lung density at month 12 (HU)	-663.3 ± 77.6	-663.3 ± 77.6
Lung volume at month 12 (mL)	2695.9 ± 1088.8	2545.5 (859-5655)
∆ subcutaneous adipose tissue volume (mL)	77.4 ± 425.5	3 (-509 - 2232)
∆ mediastinal adipose tissue volume (mL)	7.43 ± 16.0	4.5 (-77-50)
∆ lung density (HU)	12.56 ± 22.3	13.45 (-122-196)
∆ lung volume (mL)	-101.9 ± 631.2	-41 (-2481-2794)

HRCT: High-resolution computed tomography, BMI: Body mass index, ∆: Change from diagnosis to month 12.

**Table 2:** Analysis of the demographic data and semi-quantitative values measured on HRCT according to the presence of progression

	No progression (n= 23)	Progression present (n= 34)	
	n (%)	n (%)	p
Gender			
Female	8 (34.8)	14 (41.1)	0.857
Male	15 (65.2)	20 (58.9)	
Mortality	7 (38.9)	11 (61.1)	0.005**
Pirfenidone	11 (47.8)	20 (58.8)	0.234
Nintedanib	12 (52.2)	14 (41.2)	

HRCT: High-resolution computed tomography, *p< 0.05, **p< 0.001, a: Chi-square and Fisher’s exact tests, b: Mann-Whitney U test, c: Independent Student’s t-test, ∆: Change from diagnosis to month 12.

**Table 3:** Relationship between patients' weight or body mass index and mediastinal adipose tissue volume at the time of diagnosis"

	Mediastinal adipose tissue volume at diagnosis
		r	p
Weight (kg)	0.116	0.312
BMI (kg/m^2^)	0.120	0.297

*p< 0.05, Spearman correlation test, BMI: Body mass index.

**Table 4:** Univariate and multivariate Cox regression analyses of the contribution of sex, age, and HRCT measurements at diagnosis to progression

	Univariate	Multivariate
			Odd ratio	95% CI	p	Odd ratio	95% CI	p
Gender			1.083	0.637-1.841	0.769	0.978	0.507-1.886	0.947
Age	0.998	0.970-1.027	0.877	0.986	0.952-.1020	0.986
Subcutaneous adipose tissue volume at diagnosis (mL)	1.000	1.000-1.000	0.693	1.000	1.000-1.000	0.524
Mediastinal adipose tissue volume at diagnosis (mL)	0.993	0.987-0.999	0.023	0.991	0.984-0.997	0.005
Lung density at diagnosis (HU)	0.998	0.994-1.001	0.232	1.000	0.996-1.004	0.979
Lung volume at diagnosis (mL)	1.000	1.000-1.000	0.250	1.000	1.000-1.001	0.100

**Table 5:** Univariate and multivariate Cox regression analyzes examining the contribution of gender, age, progression at 12th months, and HRCT measurements at diagnosis to mortality

	Univariate	Multivariate
			Odd ratio	95% CI	p	Odd ratio	95% CI	p
Gender			1.056	0.471-2.371	0.895			
Age	1.027	0.981-1.076	0.254	1.032	0.985-1.080	0.184
Progression at month 12	8.385	2.816-24.969	<0.001	6.516	1.594-26.639	0.009
Subcutaneous adipose tissue volume at diagnosis (mL)	1.000	0.999-1.000	0.069	0.999	0.999-1.000	0.054
Mediastinal adipose tissue volume at diagnosis (mL)	0.974	0.959-0.989	0.001	0.993	0.975-1.011	0.453
Lung density at diagnosis (HU)	0.999	0.994-1.004	0.623			
Lung volume at diagnosis (mL)	1.000	0.999-1.000	0.131	1.000	0.999-1.000	0.244

## DISCUSSION

The aim of this study was to investigate the effect of mediastinal adipose tissue, namely an extrapulmonary structure, on progression. We determined that mediastinal adipose tissue volume at the time of first diagnosis was more prominent in cases without progression at the 12th month compared to those with progression. There are studies suggesting that excess adipose tissue, especially subcutaneous and visceral adipose tissue is paradoxically protective in many diseases ( 7 , 8 , 9 , 10 ). Estimates of the rate of imaging progression of interstitial lung abnormalities vary, ranging from 30% over a two-year period in the National Lung Screening Trial to 48% over a five-year span in the AGES-Reykjavik study ( 8 ). Similarly, in the current study, the mortality rate significantly increased in the patients with progression at the 12th month. Moreover, increased mortality was evident in patients with early progression.

According to Putman et al. ( 10 ), patients exhibiting subpleural reticular changes, lower lobe dominant changes, diffuse reticulation, or traction bronchiectasis demonstrated a more than six-fold increase in progression rates compared to those with interstitial lung diseases (ILDs) lacking these imaging features. In similar studies, structural changes in the lung have been reported to offer insights into the progression. In these studies, the paradoxical balance of immune modulatory cytokines, such as adiponectin, leptin, acylation-stimulating protein, and interleukin-1 beta synthesized from adipose tissue has been investigated in terms of its effects on the immune system. However, upon reviewing the literature, we did not come across any similar study that provides data on IPF. The greater release of these cytokines may also be protective against IPF in patients with larger mediastinal adipose tissue volumes. In the Framingham Heart Study and AGES-Reykjavik cohorts, this increase in mortality was most strongly associated with the progression of ILDs on imaging. In the AGES-Reykjavik cohort, ILDs were associated with increased respiratory mortality, as well as increased all-cause mortality ( 8 ). In this study, unlike the literature, we investigated the effect of mediastinal adipose tissue volume at the time of diagnosis on progression and mortality. We determined that lower mediastinal adipose tissue volume at the time of diagnosis had a statistically significant effect on progression. In light of the data obtained from our study, in addition to the prevalence of bronchiectasis and reticulation already described in the literature, mediastinal adipose tissue volume measured at the time of diagnosis can also be used as a prognostic factor in predicting the risk of progression, as well as mortality. While comprehensive oncologic and cardiac studies have been undertaken regarding this topic, there is currently no study in the literature focusing on IPF ( 11 , 12 , 13 , 14 , 15 ). Moreover, the variation in mediastinal fat tissue volume between the initial diagnosis and the 12th month was also assessed in our study. Based on the data we acquired, a more substantial increase in mediastinal adipose tissue volume was observed in patients with progression. Previous studies have suggested that the rise in mediastinal adipose tissue volume was secondary to reduced lung volume. In accordance with the literature, in our study, we found that mediastinal fat tissue volume increased in patients with progression and a more significant decrease in lung volume. Therefore, conducting multicenter studies on IPF with the participation of a large number of patients is advisable. Although the mediastinal fat tissue volume is calculated automatically in the value ranges determined at the workstations, it is often included in the measurement of other non-targeted soft tissues. Therefore, the intervention of radiologists is required. This causes workload and hinders its use in routine applications.

One of the most consistent findings concerning IPF is their association with increased mortality. The presence of honeycomb, traction bronchiectasis, and reticulations in patients with IPF has been utilized as an indicator of mortality ( 16 , 17 ). Mortality is often not feasible as an endpoint for diseases presenting with chronic progressive fibrosis therefore changes in disease extent on HRCT represent a potential means of assessing treatment response ( 18 , 19 , 20 ). However, to attain a more definitive conclusion in terms of mortality, there is a need for studies with a high number of cases, in which other comorbidities that often accompany IPF are also evaluated.

In current studies in the literature, the loss of lung volume, considered to be secondary to fibrosis, is frequently observed during the progression of IPF ( 21 , 22 , 23 ). An increase in the amount of soft tissue due to fibrosis increases the mean lung density and reduces its skewness ( 24 , 25 ). HRCT density measurements have been used to semi-quantitatively assess the lung structure in a range of lung diseases, especially emphysema ( 25 , 26 , 27 ). The clinical significance of areas of high attenuation and increased density is limited due to a large number of technical and patient-related factors, such as scanner variation, inadequate inspiration, obesity, and pulmonary atelectasis ( 28 , 29 , 30 ). In IPF, a common HRCT finding is more significant volume loss in the lower lobes of the lungs. However, due to the coexistence of combined pulmonary fibrosis and emphysema, the total lung volume may increase or remain normal, albeit rarely ( 31 , 32 , 33 , 34 ). Consistent with the literature, in our study, the increase in density was statistically significantly higher and the volume loss decreased more significantly in patients with progression. The rationale for the observed statistical significance of the increase in mediastinal adipose tissue volume, compared to the other two parameters, might be linked to the aforementioned factors that restrict density increase and volume loss. However, the sensitivity and specificity of lung volume and density at the time of diagnosis were significantly lower in predicting progression at the 12th month ( Figure 4 ).

This study had some limitations. The main ones are the following: 1) While the study excluded patients with pre-existing comorbidities known to impact mortality and who were consequently undergoing treatment, the retrospective nature of the study precluded conducting a comprehensive clinical assessment of these patients in terms of comorbidities; 2) While it has been demonstrated that two distinct antifibrinolytics are not superior to one another, the utilization of different drugs within the patient group may still be regarded as a limitation ( 35 ); 3) The single-center nature of our study constitutes a limitation in itself; 4) While measurements are conducted automatically, operator intervention is necessary, particularly for measurements involving subcutaneous and mediastinal fat tissue. Another limitation of the study is the lack of evaluation regarding interobserver usability; 5) The small number of patients is a limitation in terms of the generalizability of the study.

## CONCLUSION

In conclusion, it was determined that mediastinal adipose tissue could be used as an objective parameter to predict progression in patients with IPF at the time of diagnosis and during follow-up. In addition, mortality was significantly increased in patients with early progression.

## Ethical Committee Approval

This study was approved by the Adana City Training and Research Hospital Clinical Research Ethics Committee (Decision no: 1974, Date: 09.06.2022).

## CONFLICT of INTEREST

The authors declare that they have no conflict of interest.

## AUTHORSHIP CONTRIBUTIONS

Concept/Design: HA, ÖED

Analysis/Interpretation: HA, ÖED

Data acqusition: HA, ÖED

Writing: HA, ÖED

Clinical Revision: ÖED, HA

Final Approval: HA, ÖED
